# New gantry angle‐dependent beam control optimization with Elekta linear accelerator for VMAT delivery

**DOI:** 10.1002/acm2.14598

**Published:** 2025-01-08

**Authors:** Adriaan Abraham van Appeldoorn, Johannes Gerardus Maria Kok, Jochem Willem Heiko Wolthaus

**Affiliations:** ^1^ Department of Radiotherapy University Medical Center Utrecht Utrecht Netherlands

**Keywords:** 2T and 2R, beam physics, beam symmetry, Elekta accelerators, linac lookup table, radiotherapy, servo mechanism, VMAT

## Abstract

**Introduction:**

This paper describes a method to improve gantry‐dependent beam steering for Elekta traveling wave linear accelerators by applying the measured and filtered beam servo corrections to the existing lookup table (LUT). Beam steering has a direct influence on the treatment accuracy by affecting the beam symmetry and position. The presented method provides an improved LUT with respect to the default Elekta method to reduce treatment delivery interruptions. These interruptions are known to contribute to unwanted intrafraction motion and longer treatment times.

**Methods:**

Compared to the default method of the manufacturer, this new method takes both clockwise and counterclockwise rotation to compensate for magnetic hysteresis as well as previous configuration and noise filtering into account. The improved method to determine the lookup table uses service graphing information from the linac without the need for additional symmetry information. The clinical configuration of the flattened beam energies remains untouched during the data record.

**Results:**

This method results in a configuration where the gantry‐dependent steering is optimized over the full arc with optimal balance in the hysteresis and minimizing the effect of errors in the steering values. This method is a less error‐prone process compared to the methodology described in previous research, still achieving a reduction of interruption of about 60 percent compared to the Elekta method.

**Conclusion:**

This study shows a simplified way to optimize linac stability with improved LUT. The optimized LUT results in a lower number of interruptions, preventing downtime, and a lower risk of intrafraction motion due to longer treatment time.

## 1 INTRODUCTION

Modern treatment techniques, like IMRT and VMAT, push the steering abilities of conventional linear accelerators (linac) to the limits of their internal tolerances. For safety reasons, radiation beam interruptions occur if deviation in beam steering exceeds the system tolerances. The beam steering system of the linac accounts for changes in magnetic fields experienced by the beamline. These magnetic fields depend on changing magnetic fields in their neighborhood, for example, due to adjacent MRI systems.^1^, ^2^ Correct beam steering means the electron beam on the right place and right angle on the target, resulting in a well‐positioned and symmetrical radiation beam. For the Elekta system studied here, a beam interruption occurs if the measured asymmetry (or tilt) exceeds 5% or the associated servo steering current exceeds 50 mA, which is a set by the manufacturer.

Generally, these types of treatment interruptions can be resumed after reset by authorized persons (e.g., RTT, physicist, or engineer). The clinical impact of steering‐related interruptions is the extension of overall treatment times, thereby increasing the effect of intrafraction motion or, in extreme cases, even leading to treatment delay.

The default manufacturer's method to calibrate the gantry‐dependent steering uses data from a single gantry rotation direction only and has no additional data processing to remove noise or outliers. Previous research^3^ showed a reduction of beam interruptions for the Elekta SL25 traveling wave linear accelerators (Elekta AB, Sweden). This was achieved by including both rotation directions and smoothing in the correction. In that research, also an additional input factor had to be included, and beam symmetry steering servo mechanisms had to be disabled. This new study describes a simplified method based on servo mechanism contribution; however, without the need for an additional input factor therefore being more efficient, less risky, simpler, and more equivalent to the default method of the manufacturer. Unlike the default method, this method includes background information on both rotation directions, previous control parameters (to account for periodic magnetic changes in the environment), and mathematical data fit to avoid anomalous or missing data points that may occur in practice.

Symmetry of the radiation beam is monitored with the ion chamber mounted in the radiation head of the linac. This ion chamber has several electrodes, two of which are arranged such that the signal difference between the electrodes reflects the degree of asymmetry in the beam (tilt). This tilt is called “2R error” (Radial) for inline and “2T error” (Transverse) for crossline direction.

**FIGURE 1:**
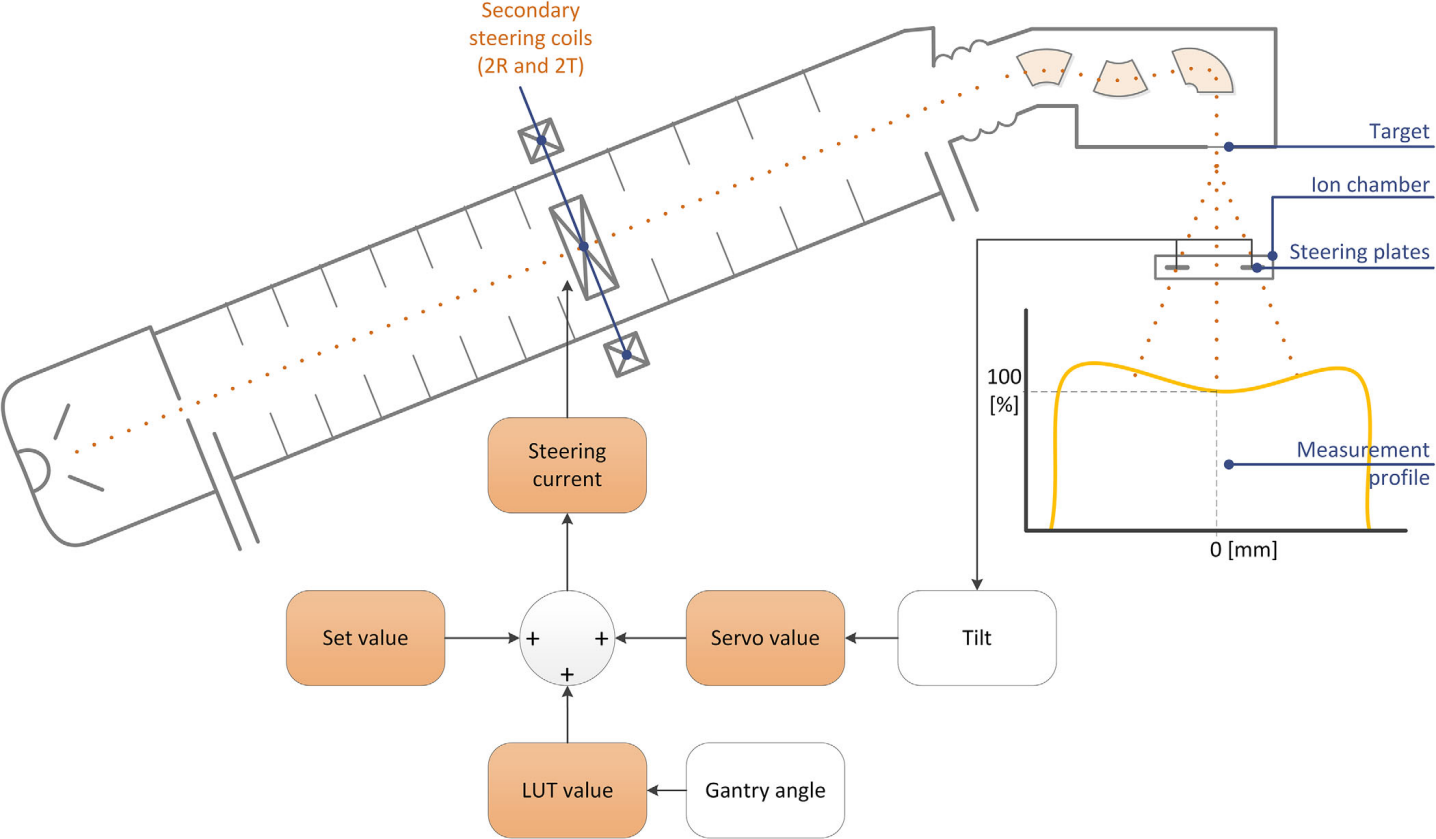
A simplified graphical overview of the steering mechanism of the Elekta linear accelerator (Elekta AB, Sweden). A secondary set of two steering coils (2R and 2T) are located halfway down the waveguide (accelerating structure). With the correct current through these steering coils, the electron beam is aligned such that the target is hit at the correct angle and a symmetrical radiation beam is produced. The actual steering currents are set by three parameters: The base value (Set value), a LUT, and the value of the servo mechanism. The LUT is the primary correction to compensate for the external magnetic distortion per gantry angle. The servo mechanism applies a secondary correction by controlling the coil current based on the readout of the steering plates of the integrated ion chamber. 2R, "2R error” (Radial); 2T, “2T error” (Transverse); LUT, lookup table.

The control mechanism for the 2R‐2T secondary beam steering current requires three input parameters and is shown in Figure 1. The first parameter is a static “Set value” of the 2R‐2T current to aim for. Secondly, a correction for the external magnetic field variation per gantry angle, which is stored in a LUT. This LUT has 128 positions that represent the full potentiometer range of the readout system, leading to a less than a hundred positions for the full clinical arc of 360 degrees. When the gantry of the linac rotates, the vector of the magnetic field changes, as well as the linac magnets and metal construction that influences its residual magnetic field. These changes in the magnetic field are static per gantry angle for the LUT configuration. As every position in the LUT represents a part of the gantry arc (about four degrees), plotting the LUT values will show a sinusoidal shape due to the circular rotation of the gantry against the dominant surrounding magnetic field vector (typically related to the geomagnetic field).

The third contribution is the “servo value”, which is derived from the ion chamber tilt signal. This active component compensates for ‘slow’ non‐deterministic effects by adapting the steering current such that the error based on the readings of the ion chamber steering plates is minimal.

The three parameters, Set value, LUT value, and servo value, can be retrieved using the service mode of the linac control software (Integrity 4.0).

Besides the slow non‐deterministic effects, there is also a small difference in the compensating effects for the clockwise (CW) and counter‐clockwise (CC) gantry directions due to magnetic hysteresis. This hysteresis is based on different contributions like previously applied steering currents, magnetic linac components (type of steel, ion pumps, and magnetron), Earth magnetic field, and nearby MRI systems. As a result, the errors observed during CC rotation are higher using the manufacturer's default method. Creating an LUT based on data from a single moment may result in an unbalanced LUT, which may lead to degradation compared to the existing LUT. The inclusion of steering data with information about both directions of rotation, previous control parameters, and applying filtering, these shortcomings can be overcome.

This study describes a new robust method to improve the linac beam steering lookup tables and provides example results demonstrating a more stable radiation beam.

## 2 METHODS

The steering current (I_steering_) is the sum of the Set value and the scaled LUT and servo values (Equation 1). The servo value is zero at beam start. Thereafter, any detected asymmetry (tilt) causes the servo Value to be non‐zero, thus changing the sum of the three inputs. This new I_steering_ value changes the trajectory of the beam path, producing a new tilt signal, and so on until the tilt signal is zero.

(1) $$\begin{array}{ccc} \;I_{steering} & = & Set\;value+LUT\;value*\frac{Calibration\;gain}{2048}\\ & & +\;Servo\;value*\frac{Calibration\;gain}{2048} \end{array}$$

If these first two parameters of the control mechanism (Set value and LUT) together lead to a deviation from the desired I_steering_ value, there is an increased chance of beam interruptions during treatment due to symmetry errors.

The default LUT‐creating method, as described in the Elekta manuals,^4^, ^5^ is sensitive to noise and spurious readings. In this procedure, the gantry rotates CW from −183° to + 183° during beam on. The control system sets up the gantry‐dependent beam control values by storing the feedback values ​​from the servo mechanism in the corresponding LUT.

**FIGURE 2:**
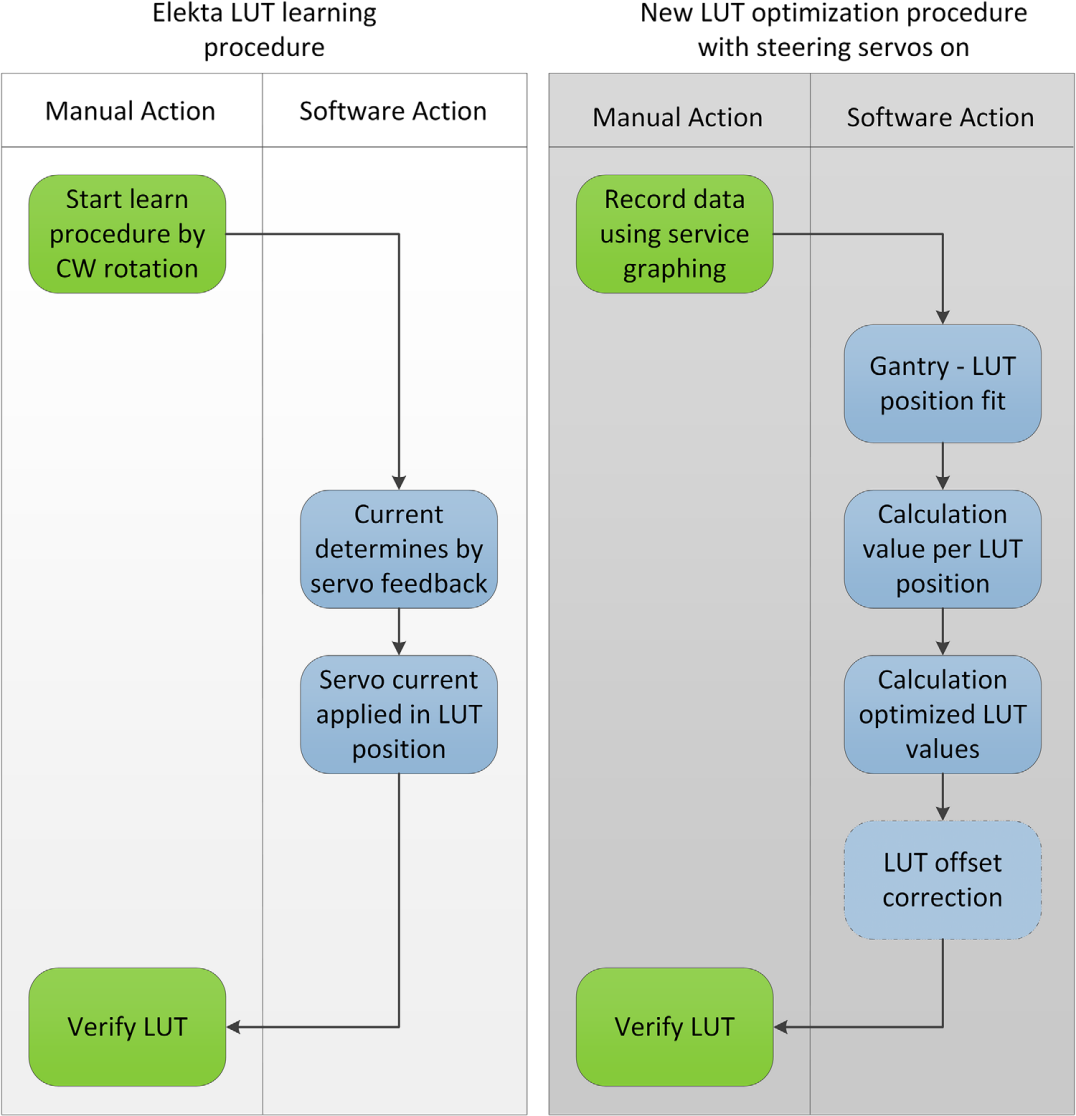
Flowcharts of the Elekta and newly proposed method to acquire a LUT for gantry‐dependent beam steering. The default Elekta procedure takes fewer dependencies into account compared to the new method. LUT, lookup table.

The improved method to generate the beam steering LUT extends the Elekta default procedure but is simplified compared to previous work.^3^ Figure 2 shows a comparison of the default Elekta procedure with the newly proposed procedure.

### 2.1 Creating data using the service graph

As input for the presented LUT optimization method, a dataset of 2R and 2T current control values are required, which is the confirmed value of steering current (I_steering_), actual LUT position, actual LUT value, and the servo value (Table 1). A 40 × 40 cm^2^ open field is used, and the dose rate is fixed to half of the maximum. This dataset is recorded using the service graph functionality in the linac control software (Integrity) at the maximum sampling frequency (4 Hz) for a full arc of CW and CC gantry rotation. Noise is reduced by recording multiple measurement samples for each LUT position (between four and seven samples, depending on the gantry rotation speed). To ensure sufficient data points, the gantry rotation speed is reduced by a temporarily low automatic system unit (ASU) speed and includes samples outside the clinical gantry angle range (±183 degrees).

**TABLE 1:** Items and part numbers settings that are used to record the input LUT for our method.

	Item	Part	Description
*X*‐axis:	148	4	Gantry angle confirmed value
*Y*‐axis:	164	4	2R I control confirmed value
		153	2R I control LUT position on specific gantry angle
		209	2R I control servo mechanism value, integrity 4+
		211	2R I control LUT value on specific gantry angle, integrity 4+
	165	4	2T I control confirmed value
		153	2T I control LUT position on specific gantry angle
		209	2T I control servo mechanism value, integrity 4+
		211	2T I control LUT value on specific gantry angle, integrity 4+

Abbreviations: 2R, “2R error” (Radial); 2T, “2T error” (Transverse); LUT, lookup table.

Before starting the actual data recording, a beam warmup of approximately ten seconds using a 40 × 40 cm^2^ field is applied to obtain a stable radiation beam. Recording the data takes about five minutes. Afterward, the data is exported as a service graph (.xml) to a network drive. With a developed MATLAB® (The MathWorks, Inc., Natick, USA) application, the recorded data is processed to create an optimized LUT.

### 2.2 Processing the data

First, the position in the lookup table is mapped to the gantry angle, yielding an average gantry angle (generally halfway) for each discrete LUT position. Subsequently, the servo‐based (correction) value and the actual value (LUT_actual_) for each LUT position are determined. To derive an optimal LUT, applicable to both rotation directions, the combined (average) servo contribution includes the measured data of both rotation directions (which differs due to hysteresis).

To maintain consistency in clinical linac behavior, reducing effects of day‐to‐day measurement variation (e.g., magnetic field fluctuation at the moment of record) and preventing over‐correction, only a fraction of the full correction is applied to the actual existing LUT as shown in Equation 2.

(2) $$\begin{array}{c} \;LUT_{raw}=LUT_{actual}+combined\;servo\;value*fraction \end{array}$$

This fraction value can be used to balance between new and existing data. Note that a full correction (i.e., fraction = 1) is used initially for a new installation or when a systematic change in the LUT is expected.

To avoid the propagation of noise spikes in the new LUT, the LUT_raw_ data (Equation 2) are fitted to a sinusoidal fit function as a function of the gantry angle, consistent with the theoretically expected behavior due to the gantry rotation. The fit function model consists of a sum of five sinusoids to account for the inhomogeneity in the magnetic field. In addition, the fit function model allows extrapolation of the data over angles beyond those that were acquired. This will result in LUT values at all clinical gantry angles, even if the input data do not contain all gantry angles.

To obtain optimal functionality for regular beam tuning on gantry zero, the new LUT data can be normalized with a set value equal to the observed I_steering_ with the gantry at 0 degrees.

## 3 RESULTS

**FIGURE 3:**
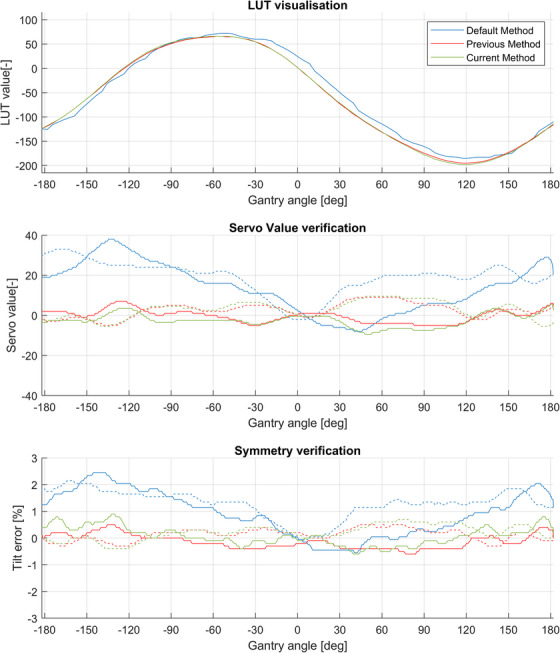
An example of the effect of the methods to create a beam steering LUT. These verification graphs are normalized at gantry angle zero to compare only the gantry dependency, where the solid line is in CW‐ and the dashed line in CC direction. The graph on top show the applied LUT for each method. In the middle, the required servo values are shown. It can be seen that the default method, shows the most variation. At the bottom, the graphs show a verification with steering servos disabled, whereas the default method shows a more variable tilt. Beams can expect such a tilt for each gantry angle at startup of the beam due to the LUT. The input for optimization, the servo value shows a proportional relation to the Tilt error which is used as input for optimization in previous work.^3^ The “Previous method” (red) and “Current Method” (green) shows a broad similarity, in all the three subgraphs and improved behavior in the tilt or servo current. CC, counter clockwise; CW, clockwise; LUT lookup table.

Results of the described procedure are compared to results of the default Elekta method (“Default Method”) and results based on the procedure of previous work^3^ (“Previous Method”). The results from this study, with the servo mechanisms enabled, are called the “Current Method.” These results are shown in Figure 3.

These results of the previous and current method are very similar regarding the servo value and symmetry. The previous method had shown a reduction of about 60 percent of beam interruptions^3^ and based on similar symmetry and the servo values over all the gantry angles, the reduction of interruptions for the current method did not change.

## 4 DISCUSSION

We have described a method of generating a LUT that provides operation with reduced beam interruptions. This result is obtained by reducing the effect of linac calibration and ambient magnetic fields from the data acquisition process. Our implementation of this technique shows an approximate 60 percent decrease in symmetry‐related beam interruptions during treatment deliveries.

## 5 COMMENTS FOR PRACTICAL CLINICAL IMPLEMENTATION

The method to create hysteresis‐compensated and noise‐free lookup tables is only valid when both servo mechanisms are switched on. Older linacs, as well as new systems for the flattening filter free (FFF) beams, have the 2T servo switched off by default. The servo system should then be switched on temporarily prior to performing the presented LUT procedure and needs to be switched off later before clinical use of the linac.

At the time of the previous study,^3^ the linac control system could not record the LUT values and servo values with service graphing according to Table 1. The current method eliminates the need for an additional input variable, such as tilt error correlation. Therefore, this method is not impacted by the specific linac calibration and surrounding magnetic field. Compared to the previous method, which experienced a delay in the tilt data of the ion chamber due to filtering, the current input (servo value) has no delay in the readout. Consequently, no data shifting is necessary for the current method. In summary, this method is faster to perform and less error‐prone compared to the default method and the previous method where an additional input parameter is required.

These data should be recorded with a single fixed (not varying) dose rate to avoid any other dependencies or correlations than to gantry angle. According to the current Elekta manual,^5^ it is advised to perform (most) beam‐tuning procedures at half of the maximum dose rate. If the system is used for dynamical treatments with variable dose rates, such as VMAT, the beam symmetry behavior should be verified with consideration of all dose rates by adjusting the gantry averaged beam tilt using the set value. Due to magnetic field fluctuation, it is recommended to avoid overcompensation by applying a correction factor as mentioned in the Method section. In the long‐run, it is seen that fewer adjustments were needed.

According to the previous work,^3^ suboptimal beam steering has just a limited effect on the dose deposition, especially when the servo mechanisms are enabled. The real benefits can be found in the reduction of interruptions on the linac for the clinic, where the previous work has shown a reduction of the number of interruptions of approximately 60 percent. However, delivery improvements can be expected for treatment plans with a high number of segments with low dose, with high variation in dose rate, and for gating where a correct beam startup will have a significant effect on the total dose delivery.

After applying the new LUT, gantry‐dependent verification should be performed. If multiple energy configurations (e.g., FFF and electron energies according to the Elekta manual^5^) are using the same LUT, dose delivery for these energy configurations has to be verified as well. This verification can be done by verifying the gantry‐dependent steering and symmetry parameters from the linac with the service graphing functionality or using an external QC device, like a 2D panel dose detector mounted on the gantry head.

Using this LUT procedure, incorporation of linac stability and dosimetry parameters or optional external QC devices is possible. This will help prevent downtime and optimize existing QA procedures. Utilization of these combined parameters and tools will result in a time‐efficient QC session, a good check of the machine performance, and a useful reference. Most importantly, this can further reduce the number of beam interruptions during clinical use.

## 6 CONCLUSION

This study describes a simplified method to improve beam steering by deriving an optimal lookup table. The new method is faster, less error‐prone, and has fewer dependencies on other linac settings. As a result, the linac stability is increased in terms of reduced beam interruptions. Similar to previous work,^3^ this method reduces downtime and extension of the treatment time caused by these beam interruptions. The clinical benefit of shorter treatment times relates to the reduced intrafraction motion of the target volume.

## AUTHOR CONTRIBUTIONS

Adriaan Abraham van Appeldoorn developed the theory and performed the measurements and analysis. Johannes Gerardus Maria Kok and Jochem Willem Heiko Wolthaus verified the analytical methods and contributed to the design and implementation of the research. Jochem Willem Heiko Wolthaus supervised the findings of this work. All authors discussed the results and contributed to the final manuscript.

## CONFLICT OF INTEREST STATEMENT

The authors declare no conflicts of interest.
